# *Magnetotactic* Bacteria and Magnetosomes as Smart Drug Delivery Systems: A New Weapon on the Battlefield with Cancer?

**DOI:** 10.3390/biology9050102

**Published:** 2020-05-19

**Authors:** Danuta Kuzajewska, Agata Wszołek, Wojciech Żwierełło, Lucyna Kirczuk, Agnieszka Maruszewska

**Affiliations:** 1 Institute of Biology, University of Szczecin, Felczaka 3c St, 71-412 Szczecin, Poland; danusia.kuzajewska@o2.pl (D.K.); lucyna.kirczuk@usz.edu.pl (L.K.); 2 Department of Medical Chemistry, Pomeranian Medical University, Powstańców Wlkp. 71 St, 70-111 Szczecin, Poland; wojzwi@pum.edu.pl

**Keywords:** *Magnetotactic* bacteria, magnetosomes, drug delivery systems, cancer, targeted therapy

## Abstract

An important direction of research in increasing the effectiveness of cancer therapies is the design of effective drug distribution systems in the body. The development of the new strategies is primarily aimed at improving the stability of the drug after administration and increasing the precision of drug delivery to the destination. Due to the characteristic features of cancer cells, distributing chemotherapeutics exactly to the microenvironment of the tumor while sparing the healthy tissues is an important issue here. One of the promising solutions that would meet the above requirements is the use of *Magnetotactic* bacteria (MTBs) and their organelles, called magnetosomes (BMs). MTBs are commonly found in water reservoirs, and BMs that contain ferromagnetic crystals condition the magnetotaxis of these microorganisms. The presented work is a review of the current state of knowledge on the potential use of MTBs and BMs as nanocarriers in the therapy of cancer. The growing amount of literature data indicates that MTBs and BMs may be used as natural nanocarriers for chemotherapeutics, such as classic anti-cancer drugs, antibodies, vaccine DNA, and siRNA. Their use as transporters increases the stability of chemotherapeutics and allows the transfer of individual ligands or their combinations precisely to cancerous tumors, which, in turn, enables the drugs to reach molecular targets more effectively.

## 1. Introduction

An enormous challenge for modern medicine is the increased incidence of cancer and high mortality among cancer patients. According to the GLOBOCAN data for 2018, over 18 million new cancer cases and almost 10 million deaths due to these diseases have been estimated to have occurred [1]. The late stage of the disease, the development of multi-drug resistance by tumor cells, the location of tumors, as well as the side effects of therapy, often make conventional forms of treatment ineffective or impossible to implement. Therefore, there is a need to develop new therapeutic strategies that would increase the effectiveness of treatment as well as improve the quality of patients’ lives [2,3,4].

One of the new research directions in this area is the design of effective distribution systems for therapeutic compounds [5,6]. One of the reasons for the failure of chemotherapy is the development of multidrug resistance (MDR) by cancer cells, which is facilitated by several mechanisms. Some of these mechanisms prevent the drug from achieving its molecular target. This is due to: (a) the reduced absorption of the drug, which occurs by diffusion or endocytosis, as well as by the participation of receptors, (b) the activity of certain cell membrane proteins that actively export compounds outside the cell, (c) the inactivation of drug molecules by the enzymes of xenobiotic detoxification of the system, and (d) sequestration within the cell [7].

In the case of solid tumors, the issue of drug delivery to the area of cancer tissue is no less important. The efficiency of drug molecule transport is limited by many biological barriers, affecting, among other things, the pharmacokinetics and solubility of the compound. These include: (a) interaction with blood serum proteins (some chemotherapeutics may be subject to opsonization or enzymatic degradation), (b) the activity of the cells in the phagocytic system, (c) the permeability of blood vessels regulated by the endothelial structure, (d) the varying degree of vascularization and tumor size, affecting distribution gases, nutrients, and metabolites, and (e) the complexity of the microenvironment within the tumor (e.g., extracellular matrix protein activity) [8,9].

The very nature of cancer cells is also a significant issue. Their phenotypic and genotypic features allow unrestricted growth and invasiveness not subject to external control, and, at the same time, they are not specific enough to be a precise target for therapeutic compounds. As a result, many classic chemotherapeutic substances are not selective and also have cytotoxic effects on normal cells [10].

In the context of the above-mentioned problems, the key issue is, therefore, to develop therapeutic strategies in which chemotherapeutics would effectively overcome biological barriers, affecting their stability, distribution, and achieving the molecular goal [5,11]. One of the promising solutions that would meet the above assumptions is the use of *Magnetotactic* bacteria (MTBs) and bacterial magnetosomes (BMs). They are organelles isolated from *Magnetotactic* bacteria. The growing number of scientific studies on these unusual organelles indicates that due to their biological and physical properties, BMs can potentially be a tool in cancer therapy.

The idea of using MTBs and BMs in biomedical sciences, particularly in medicine, is a relatively new research problem. Review work, to date, has generally described the applicability of these microorganisms and organelles in a general manner, presenting a wide range of possible applications and solutions. More work deals with the biotechnological aspects of modifying magnetosomes. It seems that MTBs and BMs can be used not only in treatment but also in the diagnosis and monitoring of various diseases, including cancer. This review article is, to our knowledge, the latest, most detailed summary of the work that only deals with the use of MTBs and BMs as nanocarriers in cancer chemotherapy.

Our work discusses achievements in improving the therapeutic effectiveness of not only classic anticancer drugs but also new treatment strategies that use MTBs and BMs. The following sections describe the biology of MTBs and the physicochemical properties of BMs. The next sections discuss the aspects of the biosafety of their use in medical practice. The following sections discuss the current state of knowledge on the possibilities of using BMs as nanocarriers for anthracycline antibiotics, cytarabine, antibodies, vaccine DNA, genes, and a combination of chemotherapeutic agents.

## 2. *Magnetotactic* Bacteria and Their Magnetosomes

**Figure 1:**
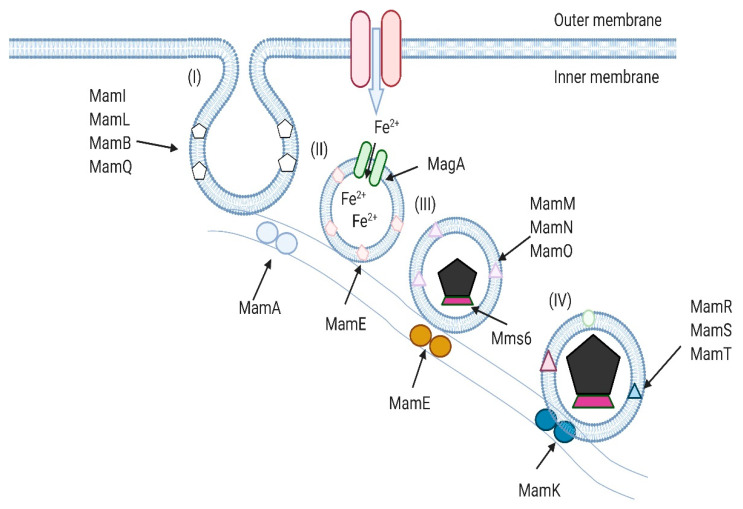
Schematic illustration of the hypothesized mechanism of magnetosome formation (MamA, -B, -E, -K, -L, -M, -N, -O, -Q, -R, -S, -T; Mms6; MagA—proteins involved in formation and maturation of magnetosomes).

*Magnetotactic* bacteria (MTBs) were discovered in 1958 by Salvadore Bellini, but works describing these microorganisms, written in 1963, were not published at the time. The discoverer of *Magnetotactic* bacteria is considered to be Richard P. Blakemore, who was the first to publish a work thereon in 1975 [12]. MTBs are a polyphyletic group of bacteria whose representatives belong to three classes of the *Proteobacteria* cluster (Alfa-, Delta-, Gammaproteobacteria), the Nitrospirae cluster, and the OP3 division. MTBs are commonly found in water reservoirs and their sediments. The distribution of bacteria in salt- and freshwater depends on the presence of appropriate conditions. The likelihood of occurrence is related to environmental conditions, such as temperature (optimum at approx. 219 °C) and pH (optimum at approx. 7). Some MTBs tolerate extreme temperatures and pH conditions, living in hot springs or heavily salted waters. The variability in the occurrence of the individual species of *Magnetotactic* bacteria is also related to the depth that defines the area of the aerobic and anaerobic zones and the so-called transition zone (aerobic-anaerobic). Oxygen and sulfide derivative gradients occurring in these zones also determine the concentration of these bacteria—they occur in both the aerobic and anaerobic zones but prefer the oxic-anoxic transition zones (OATZs) with a relatively low oxygen content [13,14]. These bacteria have developed all sorts of morphological forms: rods, commas, kernels, packets, and spiral forms. In the water, MTBs move with the help of flagella. The way the *Magnetotactic* bacteria move can be mono- or bipolar, depending on the location of the flagella. The movement of MTBs is directed because they are arranged along Earth’s magnetic field lines [15]. In the water, the orientation relative to the magnetic field (magnetotaxis) is caused by specific organelles in these bacteria—magnetosomes (BMs). BM formation processes are closely related to environmental conditions, the cell proliferation cycle, and cellular stress. BMs are organelles containing magnetic minerals (nanocrystals) surrounded by a biological membrane formed by phospholipids, glycolipids, and proteins. The process of BM formation comprises several stages: (I) vesicle formation and iron transport from outside of the bacterial membrane into the cell, (II) magnetosomes alignment in a chain, (III) initiation of crystallization, and (IV) crystal maturation (Figure 1) [16].

The proteins involved in their formation and maturation are encoded by a region called the magnetosome genomic island (MAI). The MAI consists of several operons: the highly conservative mamAB, as well as the mamGFCD, mms6, feoAB, mamXY, and mamJOE-like operons. The best known in this respect are the proteins encoded by the mamAB cluster. These include, for example, the mamE, -L, and -Q proteins that participate in BM membrane formation; the mamK and mamJ proteins that anchor the magnetosomes to the cytoskeleton filaments; the mamB protein involved in iron transport, and the proteins involved in the maturation of nanocrystals (mamE, O, T, P, and S) [17,18,19,20]. Inside the cell, BMs are most often arranged in the form of a chain (sometimes they appear in aggregates or are dispersed). BMs found in MTBs contain two different types of ferromagnetic and isostructural minerals: magnetite (Fe_3_O_4_) or greigite (Fe_3_S_4_). BMs with magnetite occur in most MTBs, while BMs with greigite occur in the bacteria that have the ability to reduce sulfates. However, there are cases where bacterial cells produce both magnetite and greigite [21]. BM crystals have a different morphology—they form elongated, cubic shapes resembling those of bullets or teeth [22]. Their sizes reach 35–120 nm in length (although crystals up to 250 nm have also been found), which corresponds to the size of a single domain with a constant magnetic field. BMs with smaller sizes exhibit superparamagnetic properties [23].

## 3. The Applicability of *Magnetotactic* Bacteria and Magnetosomes in the Treatment of Cancer

**Figure 2:**
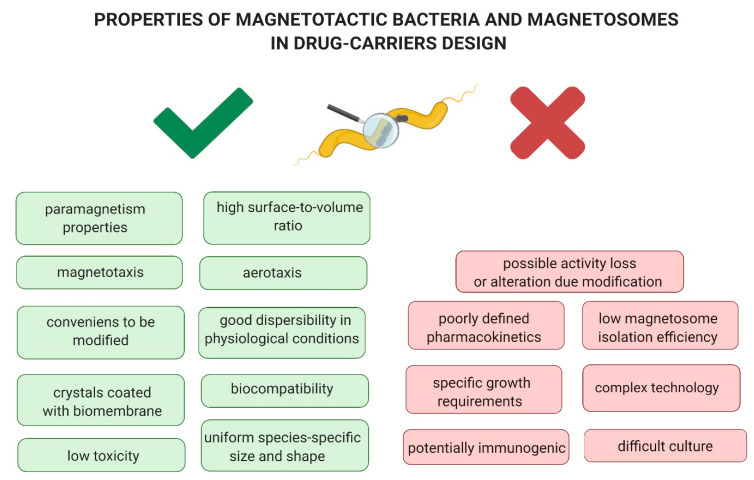
Properties of *Magnetotactic* bacteria and magnetosomes as advantages and disadvantages in drug-carriers design.

Magnetotaxis and aerotaxy, non-pathogenicity and biocompatibility, the presence of iron crystals surrounded by a lipoprotein membrane and their uniform shape and size, and paramagnetic properties and good dispersibility under physiological conditions are the unique features of MTBs and BMs that make them easy to modify (Figure 2). Properly developed, they seem to be a promising tool with an application potential in targeted cancer therapy. One of the solutions is the use of MTBs and BMs as carriers of substances with antitumor activity, also in combination with ligands that recognize molecular targets specific for the cancer cell. A therapeutic compound could be delivered entirely within the microenvironment of cancer cells, thus sparing healthy tissues. The use of molecular tools constructed in this manner would be part of a targeted therapy scheme [24,25].

### 3.1. Biosafety of Magnetotactic Bacteria and Magnetosomes

One of the major problems associated with systemic therapy (i.e., chemotherapy) is its low selectivity. Traditional anticancer drugs affect not only cancer cells but also normal cells. The necessary condition for the viability of MTBs and BMs in cancer therapy is their safety when confronted with normal cells. Therefore, in studies on MTBs, it is important to determine the pathogenicity of MTBs, as well as the cytotoxicity and genotoxicity of BMs against normal cells. Such studies were conducted both in vitro on model cell lines and cells from clinical samples, as well as in vivo on mouse models. One of them showed that BMs isolated from *Magnetospirillum gryphiswaldense* did not significantly affect the viability of the murine J774 macrophages. BM exposure also did not affect the morphology of these cells [26]. The absence of a cytotoxic effect on normal cells was similarly observed with BMs isolated from the same strain but administered to cultured mouse fibroblast L-292 line [27]. In addition to the studies on established cell lines, experiments were also carried out on donor erythrocytes. They showed that BMs from *M. gryphiswaldense* did not have hemolytic effects and that exposed red cells retained the normocyte shape [26].

BMs from *M. gryphiswaldense* have also shown no genotoxic activity. In tests performed on leukocytes (WBCs) with mitomycin as a control, it was not shown that exposure to low BMs levels caused abnormalities in the genome organization of these cells. Only at the highest concentration attempted (150 μg/mL), the chromosome breakage in WBCs was observed [26]. The lack of genotoxic and cytotoxic effects of BMs from *M. gryphiswaldense* have also been described for retinal pigment epithelia (ARPE-19) cell line and compared with the effects of synthetic magnetic nanoparticles (MNPs). Cytotoxicity tests showed that BMs, regardless of the time of exposure, did not reduce cell viability. In contrast, MNPs showed a significant cytotoxic effect on retinal cells with long-term interaction, which was additionally dependent on the concentration of the particles. In addition, DNA fragmentation analyses showed that MNPs, in contrast to BMs, induced significant DNA degradation in retinal cells. This interaction correlated with the induction of apoptosis and necrosis in the cells studied [28].

The size of a BM falling within the nanoscale allows for its very high surface-to-volume ratio. For this reason, large amounts of ligand molecules can be attached to these organelles. The biological properties of the BM membrane, in turn, allow the attachment of various bioactive factors: enzymes, genes, receptor ligands, genes, or clinically used chemotherapeutics. BM conjugation strategies that involve ligands are discussed in more detail in Sun et al. [24]. In addition, BMs are highly biocompatible. Although MTBs belong to Gram-negative bacteria, and the BM membrane contains endotoxins [29], in vivo rat and rabbit models have shown that BMs are not toxic to the body and do not cause inflammatory reactions. They also do not cause changes in the body weight, morphology, and functioning of the organs and tissues of the animals studied [30,31]. In vitro studies have also proved that these bacterial organelles as such do not show any or only a limited toxic effect on cells from the following model lines: cervical cancer HeLa [31], liver cancer H22, human promyelocytic leukemia HL60, and mouse breast cancer line EMT-6 [30]. In a rat model, BMs have been shown to be stable after their injection into the bloodstream and are not removed in the urine or feces; however, accumulation in the liver has been observed [32]. In addition, BMs have been shown to easily cross the blood–brain barrier [33]. Similarly, in studies of Qi et al. [28], it was shown that BMs were also able to cross the blood–eye barrier and increase the survival rate in relation to the ARPE-19 cell line. In addition, they induced degradation and apoptosis to a much lesser extent when compared with artificial MNPs. The slight or no effect of BMs on morphology, cell viability, and genome stability also makes them a suitable material for the construction of nanocarriers useful in the treatment of cancer. The biocompatibility of BMs can be increased by enclosing them in drops of sodium alginate, which is the polysaccharide of the cell walls of Phaeophyceae that is used to immobilize biocatalysts [34]. This concept would allow the simultaneous trapping of BM alginate capsules and drug molecules. With the additional use of a modified magnetic field, the BMs would act like particles that move to the place where the drug would be released [35]. The biocompatibility of BMs can also be increased by modifying the lipid–protein membrane, which eliminates the potential pyrogenic properties of BMs. Such modification involves coating BMs with substances, such as poly-L-lysine, citric acid, oleic acid, carboxymethylated dextran [36,37,38], chitosan, polyethyleneimine (PEI), or neridronate [39].

### 3.2. Magnetotactic Bacteria as Transport Systems

**Figure 3:**
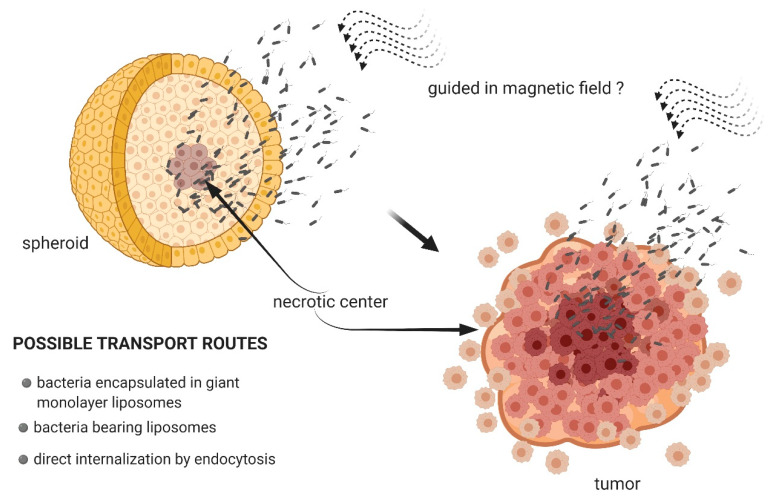
*Magnetotactic* bacteria as potential drug-carriers capable of penetrating the tumor.

There is very little literature data that would describe the use of functional MTBs as potential drug carriers. By their very nature, BMs perform this role better. Nevertheless, studies that used microfluidic systems have shown that by exploiting their natural magnetotaxes and their ability to move with the use of flagella, MTBs can be controlled with externally generated changes in the strength of the magnetic field, and this would, theoretically, also make it possible to guide these bacteria along specific paths in the human body [40]. By altering the magnetic field, it would also be possible to control their movement and introduce them precisely into the environment and the tumor. This concept makes these bacteria specialized nanorobots (Figure 3) [41,42].

Spheroids are a type of in vitro cell culture structured into a three-dimensional system. Their organization largely reflects the conditions that take place in vivo. Spheroids correspond to early, non-vascularized tumors, while their structure, similarly to neoplastic tumors, is distinguished by a gradient of oxygen, pH, and nutrients along the axis. The inner, necrotic layers of cells are areas characterized by hypoxia and low pH. In vivo, cells from these layers are resistant to chemotherapeutics [43]. It is, therefore, important to develop biocompatible drug transporters that would be able to penetrate these tumor regions. *Magnetococcus marinus* bacteria, directed in a magnetic field, have been shown to be able to penetrate spheroids constructed from adenocarcinoma LS174T cells. This is explained by their natural preference for living in niches with an oxygen gradient. This would predispose some MTBs to be drug carriers capable of penetrating cancerous tissue. However, some cellular connections could be a major barrier to MTBs and hence an obstacle to this type of strategy [44]. The encapsulation of bacteria can solve this problem. Afkhami et al. [45] described the technique of enclosing *Magnetococcus* sp. in giant monolayer liposomes. A modification of this sort would ensure better transport efficiency to the target cells. An interesting strategy in the use of MTBs as vectors is described in the work of Alsaiari et al. [46]. In this case, *M. gryphiswaldense* bacteria served as carriers for ssDNA conjugated with gold nanoparticles (AuNPs), which are also a bioimaging agent for controlling the loading and releasing processes. This model charge was effectively internalized in the bacterial cell by endocytosis. The modified bacteria were then incubated with human monocytic cell line derived from an acute monocytic leukemia patient (THP-1). Macrophages phagocytized bacteria, while ssDNA-AuNPs molecules were released by magnetic hyperthermia (heat generation caused by the induction of a magnetic field). Thus, the presence of BMs determined the possibility of releasing the charge in the tumor cell. Another interesting strategy in the use of MTBs would be their employment as indirect transporters associated with other carriers and loaded with drug particles, and they would be capable of releasing the drug directly into the cancer cells. Such carriers could be nanoliposomes combined with bacteria by the carbodiimide method, aided by the presence of free amino groups on the outer surface of the bacterial membrane, which is an excellent docking site for carboxyl residues of nanoliposomes. The attachment of nanoliposomes to MTBs increases bacterial biocompatibility [47]. The effectiveness of this solution has been confirmed in experimental conditions in vivo using a mouse model. *M. marinus* bacteria have been conjugated to nanoliposomes that contained SN-38 molecules, the active metabolite of irinotecan, which inhibits topoisomerase activity. Carriers constructed in this manner were injected into mice near the tumors induced by xenotransplantation with human colon cancer HTC 116 cells. Modified MTBs penetrated deeper into the tumors, settling in tumor areas characterized by low oxygen content. Penetration was made more efficient with the application of a magnetic field, which was used to direct the bacteria toward the tumors [48].

### 3.3. Magnetosomes as Transport Systems

**Figure 4:**
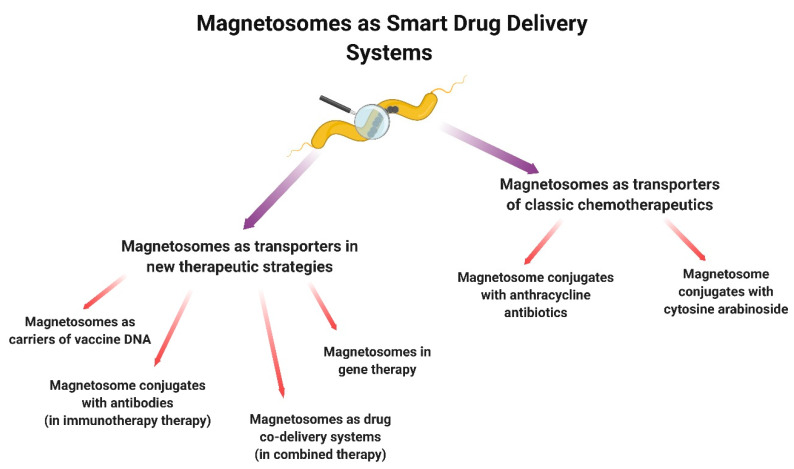
Application of magnetosomes for drug delivery in cancer therapy.

In this mini-review, we described the applications of magnetosomes for drug delivery in various areas of cancer treatment in different ways (Figure 4).

#### 3.3.1. Magnetosomes as Transporters of Classic Chemotherapeutics

##### Magnetosome Conjugates with Anthracycline Antibiotics

**Figure 5:**
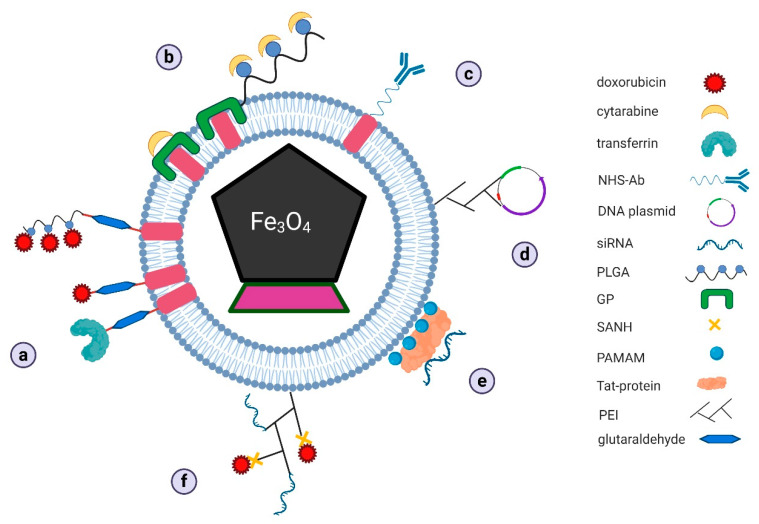
Proposed mechanisms of drug docking on bacterial magnetosomes for: (**a**) doxorubicin, (**b**) cytarabine, (**c**) antibodies, (**d**) vaccine DNA plasmids, (**e**) siRNA, and (**f**) complex of two different drugs in combined therapy (GP–genipin, NHS–N-hydroxysuccinimidyl, PAMAM–polyamidoamine dendrimers, PEI–polyethyleneimine, PLGA–poly-L-glutamic acid, SANH–succinimidyl 6-hydrazinonicotinate acetone hydrazone).

Anthracycline antibiotics isolated from *Streptococcus peucetius* (doxorubicin—DOX; daunorubicin—DAU) and their synthetic analogs (idarubicin—IDA; epirubicin—EPI) are widely used in oncological treatment. They are used in chemotherapy regimens for acute leukemia, lymphomas, myelomas, lung, breast, stomach, bladder, ovary, thyroid, and sarcoma. The mechanism responsible for their activity is complicated. They show, among other things, the ability to intercalate with DNA (aglycon is involved in this process) and stabilize the DNA-topoisomerase II complex (through the participation of a sugar residue), which consequently leads to the inhibition of DNA replication and transcription [49,50,51]. In addition, they generate reactive oxygen species (ROS), which is the reason for their significant cytotoxicity, also toward normal cells. The anthracycline pro-oxidative activity is one of the mechanisms underlying cardiotoxicity, producing serious clinical side effects [52]. To reduce the undesirable effects of anthracyclines while maintaining their antitumor activity, attempts are being made to construct new forms of these drugs (Figure 5a).

One of the promising forms of modification is the conjugation of DOX molecules with BMs, whose protein–lipid membrane is rich in amino groups NH_2_ [32]. These groups are also present in the DOX structure, but they are not responsible for the activity of the drug. The team of Guo et al. [53] showed that DOX could be bound to the BMs membrane using bifunctional compounds with cross-linking properties, such as glutaraldehyde, disuccinimidyl suberate (DSS), 3-(2-pyridyldithio)propionic acid N-hydroxysuccinimide ester (SPDP), and disuccinimidyl carbonate. It was shown that the highest conjugation efficiency was achieved with glutaraldehyde. The amount of bound DOX was 874 μg per 1 mg BMs, which corresponded to a yield of about 47%. DSS and SPDP bound the drug to a slightly less extent (784 and 624 μg/mg, respectively; 44% and 38%). The achieved values were higher than, for example, those observed in synthetically obtained superparamagnetic iron oxide nanoparticles conjugated with DOX via polyethylene glycol (520 μg DOX/mg BMs) [54]. DOX and BM conjugates were characterized by good dispersion in water and were uniform in shape and retained their magnetic properties. Interestingly, these conjugates were stable in a physiological pH buffer, and significant drug release was observed in a pH 3.5 buffer. This is an important property from the point of view of effective drug transport to a cancerous tumor, as well as of the distribution of drug particles within the tumor. It can, therefore, be expected that DOX, combined with BMs, will not be released in the lumen of blood vessels. Moreover, given the pH gradient within the tumor, the drug would easily be released from the complex only in the acidic microenvironment of the tumor. Such properties would condition less drug cardiotoxicity. In the studies described, it was also shown that BM-related DOX retained its cytotoxicity against liver cancer Hep G2 cells as well as breast cancer MCF-7 cells [53].

The same team showed that BMs bound to DOX via poly-L-glutamic acid (PLGA) exhibited similar physicochemical and cytotoxic properties [55]. Similarly, promising results were obtained in the work of the team of Sun et al. (2008) [56]. They showed that BM-related DOX was, like pure drug molecules, captured by EMT-6 cells. However, although the number of BMs in the cells correlated with the incubation time, the uptake rate was much lower than with DOX alone. The drug associated with BMs showed significant stability in solutions containing serum, and its molecules under these conditions were released slowly. In vitro cell culture studies have shown that BMX-related DOX retains cytotoxicity to HL60 and EMT6 cell lines. Moreover, both forms of the drug have inhibited the expression of the c-myc oncogene in the tested cell lines [56], whose protein product acts as a transcription factor that controls the expression of genes responsible for increased cell proliferation, angiogenesis, metastasis and of genes determining genetic instability and cell renewal potential [57].

The previous work of Sun et al. (2007) [58] showed that DOX/BMs also had cytotoxic activity on H22 cell lines. This effect was observed in a mouse model in both in vitro and in vivo studies. Both free and bound DOX caused similar tumor suppression (by 78.6% and 86.8%, respectively) with a mortality rate of 80% and 20%, respectively. Histopathological studies confirmed the lower cardiotoxicity of DOX/BMs. Reduced c-myc expression was also shown in H22 cells exposed to conjugated DOX [58].

More detailed research on this form of therapy using liver cancer cells was described in Geng et al. [59]. The authors developed conjugated forms (also with glutaraldehyde) for DOX and two other anthracycline antibiotics: daunorubicin (DAU) and epirubicin (EPI). It was demonstrated that the DOX-BM and DAU-BM conjugates were characterized by greater cytotoxicity to Hep G2 cells than their free forms, whereas the interaction was weaker with normal hepatocytes from the HL-7702 line and caused fewer morphological changes. Perhaps, this effect was associated with the differences in the rate of passage through the semipermeable membrane. In addition, analyses of diffusion in cell-free systems showed that anthracycline molecules released from the complexes diffused more slowly across the membrane into the serum solution than their free forms, indicating the ability of BMs to prolong drug release.

This work also sheds light on the mechanisms of transporting drug-BM conjugates. Due to the size of the complexes (486–760 nm), individual DOX/BMs were internalized by caveola-related endocytosis, and nanoparticle aggregates were captured by micropinocytosis. Both mechanisms shared the participation of early/late endosomes and lysosomes, and, in their course, a DOX release from the complexes and its relocation to the molecular target was observed. The DOX-BM and DAU-BM complexes also showed to induce apoptosis in both Hep G2 and HL-7702 cells by an increased expression of genes encoding p53 protein and caspase 3 and inhibition of c-myc oncogene expression, and this effect was stronger on cancer cells. In addition, anthracyclines were shown to generate ROS to a much lesser extent in complexes with BMs. In vivo analyses confirmed the anticancer effect of anthracycline-BM complexes. In Hep G2 xenografts, a reduction in tumor size was observed, while no disruption in the cardiomyocyte ultrastructure was observed [59].

The same team designed a new form of nanotherapeutic by combining BMs with DOX and transferrin (Tf)-(DOX/BM/Tf) simultaneously [60]. Tf is an iron-binding protein whose receptor (TfR, CD71) is found in nucleated cell membranes, and its increased expression is seen in cancer cells. Therefore, the TfR receptor may be a molecular target in targeted therapy [61]. Hep G2 and HL-7702 cells showed to have different levels of TfR gene expression, which explained the observed increased uptake of nanocarriers by tumor cells compared to normal cells. DOX/BM/Tf complexes showed cytotoxic activity on normal and tumor cells comparable to DOX/BMs, with cytotoxic interaction on Hep G2 cells being clearly stronger. It was also shown that drug conjugates with BMs and Tf also induced apoptosis to a much greater extent in tumor cells than in normal cells. These observations were consistent with the results of expression analysis of genes encoding p53, Bcl-2, and c-Myc proteins associated with the regulation of pro- and anti-apoptotic pathways, as well as genes encoding initiator and executioner caspases. In xenografts, DOX/BM/Tf complexes administered intravenously inhibited tumor growth much more effectively than DOX/MBs or free DOX. In addition, it was confirmed that DOX/BM/Tf complexes did not cause significant pathological changes in organs, especially in heart tissues. In light of the results described, it seems that BMs additionally conjugated to a receptor ligand, particularly expressed in cancer cells, maybe an interesting nanocarrier for classically used chemotherapeutics [60].

BMs conjugated with Tf can also be considered as an effective monitoring and treatment tool due to the ability of the cancer cells to metastasis. Recent studies indicate that circulating tumor cells (CTCs) and CTC clusters play an important role in cancer development. CTCs separated from the primary tumor and/or metastatic foci enter the peripheral blood. CTCs are thought to be responsible for metastasis and recurrence. The knowledge about these cells gives new possibilities in the field of chemotherapy, treatment monitoring, diagnostics, and prognosis of disease development [62,63]. TfR is one of the surface markers overexpressed in cancer cells [61]. It may serve as a molecular target for capturing and killing of CTCs pool [64]. It is likely that the observed efficacy of BMs conjugates with chemotherapeutic agents that have been administered subcutaneously near the tumor [58] may be limited only to the injection site. BMs-drug conjugates appear to be stable in serum and physiological pH buffers [53,56]. This means that the intravenous administration of BMs-drug complexes linked with Tf can be a better therapeutic solution due to better distribution of the nanocarriers in the body. Such a strategy may prove more effective in disease monitoring and systemic treatment of cancer [65].

##### Magnetosome Conjugates with Cytosine Arabinoside

Cytosine arabinoside (cytarabine, Ara-C) is a chemotherapeutic agent from the group of pyrimidine antimetabolites. It is used in therapeutic regimens for the treatment of non-Hodgkin’s lymphomas, acute myeloid leukemia, acute lymphoblastic leukemia, or blast crisis in chronic myelogenous leukemia. The mechanism behind the activity of Ara-C is multidirectional. The primary activity is associated with the major metabolite, 5-cytarabine triphosphate (Ara-CTP), which is the antimetabolite of deoxycytidine triphosphate. It demonstrates the ability to bind to DNA and thereby inhibits DNA polymerase activity, preventing the replication and transcription of genetic material. In addition, Ara-CTP re-inhibits deoxycytidine kinase and also induces tumor cell apoptosis by modulating the signaling pathways associated with the activity of nuclear factor κB (NF-κB) [66,67,68]. The side effects resulting from Ara-C toxicity to normal cells include myelosuppression, hepatotoxicity, gastrointestinal disorders, neurotoxicity, fever, and rash [69].

BM conjugates from Ara-C can be constructed by direct binding as well as using linker substances (Figure 5b). One of the first tested bifunctional linkers was genipin (GP). BM/GP/Ara-C conjugates have been characterized by high stability and a long-lasting (up to about 3 months) release of drug molecules from complexes [70]. Promising results were also obtained by Liu et al. by creating BM conjugates with methotrexate, which is one of the potent anti-metabolite drugs [71]. In the later work of the same team, the efficiency of direct Ara-C joining and conjugation using GP or glutaraldehyde were compared. The direct method proved to be the most effective. Nanoconjugates formed by absorbing particles on the surface of the BM membrane were obtained with greater efficiency and degree of drug loading than when using linker substances [72]. An interesting solution in the research on BM modification as drug carriers seems to be the use of two conjugating molecules. BM conjugates linked to Ara-C molecules via GP and PLGA have been described [73]. In such a construct, only genipin molecules were directly associated with BMs. They were the link for PLGA chains to which numerous Ara-C molecules were attached. This theoretically provides more binding sites for drug molecules. Expanded nanocarriers were obtained with a yield of approx. 64% and a drug loading level of 39%. They were characterized by an ability to release the drug slowly and for a long time (up to 90% in 40 days). The nanocarriers prepared in this way showed cytotoxic activity on cells from the HL60 line at higher concentrations compared with the activity of Ara-C alone. In a bioimaging analysis of cells exposed to BM conjugates with Ara-C or Ara-C alone, morphological features typical of necrosis and apoptosis were observed [73]. Interesting results are provided by another work of the same team [74]. It described BM constructs that also used both linkers, i.e., PLGA and GP. However, in this case, two types of drugs were attached to PLGA polymers simultaneously: DAU and Ara-C molecules. The encapsulation efficiency and loading level of drug molecules were, for Ara-C, 68 and 32%, respectively, while for DAU, these parameters were 36 and 18%, respectively. The greater docking efficiency of Ara-C particles affected their extended release—up to 40 days. DAU was released much faster from the BM complex (within 13 days); however, no initial drug burst was observed for any of the compounds. BM-drug conjugates showed cytotoxicity to HL60 cells at a similar level to free forms of drugs, and this effect was directly proportional to the exposure time and concentration. BM constructs with molecules that differ in structure and interaction with cells can potentially serve as rational co-transporting systems in therapeutic regimens that use several chemotherapeutics simultaneously. This could not only increase the specificity and efficiency of drug transport to the destination but also reduce the side effects of chemotherapy [74].

#### 3.3.2. Magnetosomes as Transporters in New Therapeutic Strategies

##### Magnetosome Conjugates with Antibodies

One of the still-developing forms of cancer treatment is immunotherapy, which aims to stimulate the host’s immune response directed against cancer cells. Numerous successes in the treatment of cancer using immunocompetent drugs have strengthened the position of this form of clinical treatment. One of the features of cancer cells that condition their expansion is the ability to escape suppressive signals from the immune system. It is related, among other things, to the dysregulation of checkpoints and T lymphocyte activation pathways. Immunodrugs restore the balance between signaling for co-activating and co-inhibitory receptors present on these cells and participating in their activation. On the other hand, possible adverse immune responses [75] pose a threat to this type of therapy. One of the promising antigens in immunotherapy is the 4-1BB antigen (CD137)—an inducible co-stimulatory receptor found on the surface of T lymphocytes and natural killer (NK) cells. Ligation with this receptor activates pathways that lead, among other things, to the induction of apoptosis [76].

BMs can be conjugated to antibodies (Figure 5c). The Tang et al. team (2019) [77] obtained functional BM complexes with an agonist antibody against 4-1BB. Antibody immobilization was carried out with a cross-linking bis(sulfosuccinimidyl)suberate agent. This compound provides the N-hydroxysuccinimidyl (NHS) ester moiety that conjugates antibodies to the amino acid residues of BM membrane proteins via amide bonds. This technique has been shown to bind 115 antibody molecules to one BM. BM-antibody conjugates greatly encouraged the proliferation of murine T lymphocytes, and the effect observed was stronger than in systems with the antibody alone. In addition, this effect correlated with the induction of CD8^+^ lymphocytes to secrete cytokines, such as tumor necrosis factor α (TNF-α) and interferon γ (IFN-γ). The immunostimulatory activity was also confirmed in in vivo studies with the use of animal models. In mice with tumorigenesis induced by inoculation with lung cancer TC-1 cells, an intravenous administration of BMs conjugated with an anti-4-1BB antibody and directed in a magnetic field near the tumor site resulted in a reduced tumor volume and increased chance of animal survival. In addition, BM conjugates were characterized by a much greater ability to infiltrate tumor tissue and recruit within it the tumor-infiltrating lymphocytes (TILs) than the antibodies themselves. A similar ability to infiltrate BM-antibody conjugates within tumor tissue was described in the work of the Erdal et al. team (2018) [78]. The authors constructed, using NHS, BM complexes with an anti-epidermal growth factor receptor (EGFR) antibody. An EGFR plays an important role in cancer progression: it activates signaling pathways that condition tumor cell proliferation, avoidance of death signals, and tumor angiogenesis [79]. BM-anti-EGFR complexes showed the ability to bind to breast cancer cells of the MDA-MB-231 line to a higher degree than complexes of synthetic iron nanoparticles with an anti-EGFR antibody. They also showed better distribution within the tumor.

##### Magnetosomes as Carriers of Vaccine DNA

One of the immunotherapeutic strategies is based on the administration of tumor antigens or their fragments, which, in consequence, should stimulate lymphocytes to produce a cancer response (reviewed in Ghaffarifar, 2018 [80]).

The literature describes the possibility of using BMs as vectors for “vaccine” genes (Figure 5d). The BM-DNA conjugate model was described by Xiang et al. (2007) [81]. The authors showed that PEI-coated BMs protected the DNA load against DNase. Moreover, transfection efficiency (performed on baby hamster kidney BHK-21 cells) was higher than for PEI-DNA conjugates. The efficacy of BM-DNA complexes was also confirmed in in vivo mouse model tests. In animals transfected with plasmid DNA for β-galactosidase conjugated with BMs, gene expression for this enzyme was observed to a greater extent than in individuals injected with constructs in a classic form. This effect was enhanced in the applied magnetic field [81].

The application potential of BMs as vaccine DNA carriers was also confirmed in mice immunization tests with a plasmid encoding the VP1 protein from the envelope of the hand, foot, and oral disease (HFMD) virus. In the group of mice immunized with BM-DNA complexes, there was increased production of anti-VP1 antibodies and significantly higher activation of T lymphocytes. This effect was stronger with the simultaneous application of a magnetic field. These are promising results in the context of the use of BMs as vaccine carriers [81].

This potential seems to be confirmed by the results of a work done on a vaccine preparation, which was a BM construct with recombinant DNA encoding secondary lymphoid tissue chemokine (SLC), IgG Fc fragment, and human papillomavirus type E7 protein (BM/pSLC-E7- Fc) [82,83]. Previous studies have shown that the CcL21 chemokine (responsible for the recruitment of lymphocytes and dendritic cells) and IgG synergistically increase the immunogenicity of a DNA vaccine that contains the E7 encoding plasmid (E7 protein is an etiological factor of some genital cancers) [84]. BM-conjugated pSLC-E7-Fc plasmids have been shown to be efficiently transfected into murine melanoma B16-F10 cell lines, with transfection efficiency increased by using a constant magnetic field (magnetofection). A similar relationship was demonstrated in an in vivo system using a mouse model where the subcutaneously administered preparation showed the ability to penetrate tissue, leading to the expression of the recombinant gene and synthesis of the pSLC-E7-Fc fusion protein. In a murine tumor model (using TC-1 cells), the DNA vaccine used induced a strong immune response directed against tumor cells, which manifested itself in a decrease in volume, metastasis inhibition, and prolonged animal survival. In addition, the splenocytes of immunized mice showed high and specific cytotoxic activity of T cells (CTL) responses were noted against TC-1 cells expressing E7 as opposed to B16-F10 cells lacking this. The immunogenic activity of the vaccine preparation tested was also confirmed by a histopathological analysis, which showed numerous lymphocytic infiltrates in the tumor microenvironment, whereas, in control animals treated with BMs only, no histopathological changes and no clinical side effects were noted. It should be emphasized that greater in vivo vaccine efficacy was observed when magnetic field stimulation was applied after immunization [82,83].

##### Magnetosomes in Gene Therapy

Another promising anticancer strategy is gene therapy involving, among other things, silencing oncogenes and regulating transcription factors crucial for tumor progression (reviewed in Sun et al., 2019) [85]. The main problem in this type of strategy is the difficulty in introducing therapeutic constructs into target cells. Due to the physicochemical and biological properties of BMs, these organelles can be an interesting solution to this problem as vectors (Figure 5e).

It has been shown that BMs may have application potential in gene therapy of gliomas, which belong to particularly malignant tumors in treatment. One of the molecular factors that are responsible for the significant invasiveness of gliomas and poor prognosis in therapy is EGFR [79]. Han et al. (2010) [33] developed BM conjugates with siRNA-containing plasmids (psiRNA) silencing EGFR expression in human glioblastoma U251-MG cells. BMs coated with other nanocarriers—polyamidoamine dendrimers (PAMAM) and the Tat protein—were used as the initial construct. PAMAM is characterized by their safety of use and low mass, while the Tat protein shows a better ability to effectively overcome the barrier of biological membranes [86]. Tat-BM-PAMAM-psiRNA-EGFR complexes showed the ability to inhibit the rate of glioma cell proliferation in vitro and block the cell cycle in G0/G1 phases. Transfection of cells with these complexes also induced apoptosis in glioma cells and inhibited their ability to penetrate the laminin- and collagen-containing matrix barrier. The effects observed correlated with the reduced levels of EGFR and other proteins that also participate in cell cycle regulation, apoptosis, and invasiveness, such as phosphorylated protein kinase AKT (p-AKT), proliferating cell nuclear antigen (PCNA), cyclin D1, Bcl-2 protein, metalloproteinase 2 and 9 (MMP2 and MMP9), and vascular endothelial growth factor (VEGF). In these studies, the effectiveness of gene therapy using BMs was also confirmed in a mouse model. Tat/BM/PAMAM-psiRNA-EGFR transfected U251-MG xenografts showed a decrease in tumor volume, while immunohistopathological analyses of protein expression in situ corresponded to the results obtained from in vitro analyses [33]. Other promising results using siRNA were described in the work of Dai et al. (2017) [87]. Researchers constructed BM conjugates with gene silencing siRNA as a signal transducer and an activator of transcription protein 3 (STAT3). As a transcription factor for genes that are involved in cell survival, proliferation, chemo-resistance, and angiogenesis, STAT3 participates in tumor progression mechanisms and represents a new and interesting molecular target in therapy [88]. The antitumor activity of BM complexes with siRNA/STAT3 bound via PEI was checked on a model HeLa cell line. Cell transfection was shown to inhibit their proliferation and induce programmed death by apoptosis. Moreover, it was shown that immobilizing siRNA on the surface of a BM and with the use of PEI significantly reduced the degree of complex degradation when in the presence of heparin or RNase, which is an important issue, given the low stability of RNA [87].

An equally interesting concept of using BMs in gene therapy was presented in the work of Wang et al. (2018) [89]. The authors constructed BM-plasmid complexes for the co-expression of two proteins—apoptin and cecropin B (pVAX1-VA). These proteins exhibited antitumor activity by inducing cycle blocking in G2/M phases and p53-independent apoptosis, as well as cell membrane disintegration, respectively. Moreover, gene therapy using a combination of genes for these proteins can be an interesting strategy for the treatment of numerous cancers, as cecropin B may potentiate apoptin activity [90]. The therapeutic effectiveness of transfection with a BM-associated pVAX1-VA plasmid was demonstrated, among other things, for Hep G2 cell lines. In the cells tested, apoptin and cecropin B were expressed at a higher level than in control cells transfected with a lipofectamine-associated plasmid. It was noted that BM/pVAX1-VA-transfected cells showed significantly lower viability and undergone apoptosis, which was manifested by the disintegration of the mitochondrial membrane and the activation of caspase 3 and caspase 9. The effectiveness of this therapeutic strategy was confirmed in vivo in a mouse model. After BM/pVAX1-VA transfection in Hep G2 xenografts, the reduction of tumors and the presence of TILs in the tumor microenvironment were observed [89].

##### Magnetosomes as Drug Co-Delivery Systems

One of the pioneering directions in the research of cancer treatment is the aforementioned development of co-delivery systems for two or more therapeutics in one transport system using nanocarriers. This type of strategy would allow for the precise and specific delivery of drugs that differ in physicochemical properties and mechanisms of action to cancer cells and thus be a synthesis of combined and targeted therapy [91]. The little literature data that is available indicates that BMs seem to be an interesting solution in this type of strategy (Figure 5f).

The use of BMs as co-transporting systems was described in the work of Long et al. (2018) [92], in which the simultaneous use of DOX and siRNA/STAT3 was studied. These compounds were conjugated to BMs with PEI and succinimidyl 6-hydrazinonicotinate acetone hydrazone (SANH) as a bifunctional linker. It was shown that PEI-bound DOX was released slowly, especially under the conditions corresponding to the tumor microenvironment, while the siRNA bound in the nanocomplex maintained its stability. The nanocarriers constructed in this manner were fed to a HeLa cell culture. The BM/DOX-siRNA/STAT3 complex inhibited the proliferation of HeLa cells and induced their apoptosis, and the observed effect was synergistic [92].

Promising results were also provided by the work of Cheng et al. (2016) [93]. Researchers from this team developed a construct consisting of DOX and a plasmid containing sequences for the heat shock protein promoter 70 (HSP70) as an inducible factor and for interfering shRNA that inhibited the expression of polo-like kinase 1 (Plk1) (BM/DOX-shPlk1). Plk1 is a proto-oncogenic protein involved in mitosis, which is overexpressed in many cancer cells [94]. It was shown that DOX was released from nanocarriers to a much greater extent when in elevated temperatures (43 °C). The use of a thermosensitive HSP70 promoter system in the nanocarrier also led to a dependence on thermal stress in relation to the expression of Plk1 in BM/DOX-shPlk1-transfected osteosarcoma U2-OS cells. Namely, the level of Plk1 mRNA and the level of protein in the cells examined, which after transfection were exposed to a variable magnetic field that caused hyperthermia, were significantly lower compared to systems without the applied magnetic field. In addition, a significant antitumor activity of the BM-DOX-shPlk1 complexes manifested by reduced proliferation and the induction of apoptosis in osteosarcoma cells was observed only in those systems where hyperthermia was induced [93].

## 4. Limitations in the Use of *Magnetotactic* Bacteria and Magnetosomes as Drug Delivery Systems

The growing amount of data in the literature indicates that MTBs and BMs are promising research material in biomedical sciences. However, with the current state of knowledge, one should be aware that many factors still limit their application potential (Figure 2). One of the limitations is the difficulty in maintaining the culture as well as its efficiency. While MTBs are common microorganisms, their cultivation in laboratory conditions presents many difficulties due to their poor proliferation. These bacteria have special environmental requirements related to the availability of oxygen, nutrients, and iron [13,14].

The process of the biomineralization of iron crystals and the formation of magnetosomes are subject to strict gene control [17,18,19,20]. However, they are also influenced by environmental factors, such as the availability of oxygen and iron, which may consequently hamper the obtaining of homogeneous fractions from magnetosomes [95,96]. The efficiency of the isolation of the magnetosome is also a problem [97]. To increase in vitro culture efficiency and the number of magnetosomes obtained, commercial production requires multiparameter optimization that will take into account the oxygen and nutritional requirements of MTBs [98,99,100]. Currently, only a few species of MTB have been thoroughly tested in this respect, so, perhaps, better candidates for biotechnological production should be sought among other representatives of these microorganisms [101]. An attractive solution would also be the biosynthesis of magnetosomes by bacteria that have better growth parameters in large-scale cultures, e.g., *Rhodospirillum rubrum* [102].

The use of BMs as nanocarriers of drugs is associated with the modification of their biological membrane and thus changes the properties important for their application potential (including stability or dispersibility in tissue). Therefore, it is essential to select such molecules that will not affect the stability of BM complexes with drugs, which is manifested by maintaining the value of the zeta potential [69]. These substances must also not have cytotoxic properties (e.g., glutaraldehyde).

Clinical limitations in the potential use of nanoparticles as drug carriers have been discussed in detail in Park and Na [103] and Pędziwiatr-Werbicka et al. [104]. The most important issues to consider in their design and use are biocompatibility, the stability of complexes in blood serum and target tissue, half-life, potential ability to aggregate with platelets and aggregate within tissues, potential immunogenicity, toxicity to normal cells, sizes of molecules, distribution in tissues and organs, biological barriers, tissue penetration, interaction with the phagocytic system, pathways of elimination from the body.

The results of previous studies indicate that, from a medical point of view, MTBs and BMs fit perfectly into the idea of targeted cancer therapy. However, to date, research into the use of MTBs and BMs as drug carriers concerns a small group of chemotherapeutics. They have been conducted in in vitro models using continuous cell lines and on animal models. Therefore, research is needed to consider more cell and tissue types, as well as a broader range of clinically used drugs. Moreover, at this stage, analyses that use primary cultures and intensification of research using spatial models (e.g., spheroids), which better map the interactions occurring in the tumor microenvironment, would be recommended. There is still little data that would explain the issues related to the pharmacokinetics of MB-drug conjugates and their biocompatibility. Further research should also unequivocally answer the question of whether magnetosomes significantly increase the efficiency of transported chemotherapeutics, or perhaps their function would be limited mainly to the role of a carrier that precisely delivers the active substance to cancer cells.

## 5. Summary

One of the challenges present in modern medicine is the increasing incidence of cancer, especially since conventional treatments often prove ineffective or impossible to implement. One of the novel research trajectories in increasing the effectiveness of cancer treatment is designing effective distribution systems for therapeutic compounds. *Magnetotactic* bacteria (MTBs) and their unique organelles called magnetosomes (BMs), which contain ferromagnetic crystals, have great application potential in this area. Biocompatible MTBs and BMs can be used as natural nanocarriers capable of delivering chemotherapy to the target site, i.e., the cancer cell, with great precision. MTBs and BMs can easily be modified and conjugated with ligands, such as classic anticancer drugs, siRNA, DNA, antibodies, and liposomes. In addition, the ferromagnetic properties of these microorganisms allow them to be controlled inside a magnetic field. This presents a wide range of possibilities in the development of constructs, the use of which is part of the idea of targeted cancer therapy.

## Abbreviations

Akt—protein kinase Akt; Ara-C—cytarabine; Ara-CTP—5-cytarabine triphosphate; ARPE-19—retinal cells; B16-F10—murine melanoma cells; BHK-21—baby hamster kidney cells; BMs—bacterial magnetosomes; CTL—cytotoxic activity of T cells; DAU—daunorubicin; DOX—doxorubicin; DSS—disuccinimidyl suberate; E7—human papillomavirus type E7 protein; EGFR—epidermal growth factor receptor; EMT-6—breast cancer cells; EPI—epirubicin; GP—genipin; H22—liver cancer cells; HeLa—cervical cancer cells; Hep G2—liver cancer cells; HL60—human promyelocytic leukemia cells; HL-7702—normal hepatocytes; HSP70—heat shock protein promoter 70; HTC 116—human colon cancer cells; IDA—idarubicin; IFN-γ—interferon γ; J774—murine macrophages; L-292—mouse fibroblasts; LS174T—adenocarcinoma cells; MAI—magnetosome genomic island; MCF-7—breast cancer cells; MDA-MB-231—breast cancer cells; MDR—multidrug resistance; MMP—metalloproteinase; MNPs—magnetic nanoparticles; MTBs—*Magnetotactic* bacteria; NF-κB—nuclear factor κB; NHS—N-hydroxysuccinimidyl; NK—natural killer; NPs—nanoparticles; OATZs—oxic-anoxic transition zones; PAMAM—polyamidoamine dendrimers; PCNA—proliferating cell nuclear antigen; PEI—polyethyleneimine; PLGA—poly-L-glutamic acid; Plk1—polo-like kinase 1; psiRNA—siRNA-containing plasmids; pSLC—secondary lymphoid tissue chemokine; ROS—reactive oxygen species; SANH—succinimidyl 6-hydrazinonicotinate acetone hydrazone; SPDP—3-(2-pyridyldithio)propionic acid N-hydroxysuccinimide ester; STAT3—activator of transcription protein 3; TC-1—lung cancer cells; Tf—transferrin; TfR—transferrin receptor; THP-1—human acute monocytic leukemia cells; TILs—tumor-infiltrating lymphocytes; TNF-α—tumor necrosis factor α; U251-MG—human glioblastoma cells; U2-OS—osteosarcoma cells; VEGF—vascular endothelial growth factor; WBC—leukocytes.
